# White Matter Plasticity in Reading-Related Pathways Differs in Children Born Preterm and at Term: A Longitudinal Analysis

**DOI:** 10.3389/fnhum.2019.00139

**Published:** 2019-05-08

**Authors:** Lisa Bruckert, Lauren R. Borchers, Cory K. Dodson, Virginia A. Marchman, Katherine E. Travis, Michal Ben-Shachar, Heidi M. Feldman

**Affiliations:** ^1^The Developmental-Behavioral Pediatrics Research Group, Division of Developmental-Behavioral Pediatrics, Department of Pediatrics, School of Medicine, Stanford University, Stanford, CA, United States; ^2^Language Learning Lab, Center for Infant Studies, Department of Psychology, Stanford University, Stanford, CA, United States; ^3^The Gonda Multidisciplinary Brain Research Center, Bar-Ilan University, Ramat Gan, Israel; ^4^Department of English Literature and Linguistics, Bar-Ilan University, Ramat Gan, Israel

**Keywords:** prematurity, diffusion MRI, longitudinal study, reading development, tractography, white matter microstructure

## Abstract

Children born preterm (PT) are at risk for white matter injuries based on complications of prematurity. They learn to read but on average perform below peers born full term (FT). Studies have yet to establish whether properties of white matter pathways at the onset of learning to read are associated with individual variation later in reading development in PT children. Here, we asked whether fractional anisotropy (FA) at age 6 years is associated with reading outcome at age 8 years in PT children in the same pathways as previously demonstrated in a sample of FT children. PT (*n* = 34, mean gestational age = 29.5 weeks) and FT children (*n* = 37) completed diffusion MRI and standardized measures of non-verbal IQ, language, and phonological awareness at age 6 years. Reading skills were assessed at age 8 years. Mean tract-FA was extracted from pathways that predicted reading outcome in children born FT: left arcuate fasciculus (Arc), bilateral superior longitudinal fasciculus (SLF), and left inferior cerebellar peduncle (ICP). We explored associations in additional pathways in the PT children: bilateral inferior fronto-occipital fasciculus, inferior longitudinal fasciculus, and uncinate fasciculus. Linear regression models examined whether the prediction of reading outcome at age 8 years based on mean tract-FA at age 6 years was moderated by birth group. Children born PT and FT did not differ significantly in tract-FA at age 6 years or in reading at age 8 years. Sex, socioeconomic status, and non-verbal IQ at age 6 years were associated with reading outcome and were included as covariates in all models. Birth group status significantly moderated associations between reading outcome and mean tract-FA only in the left Arc, right SLF, and left ICP, before and after consideration of pre-literacy skills. Microstructural properties of these cerebral and cerebellar pathways predicted later reading outcome in FT but not in PT children. Children born PT may rely on alternative pathways to achieve fluent reading. These findings have implications for plasticity of neural organization after early white matter injury.

## Introduction

Neuroplasticity can be defined as the ability of the brain to reorganize itself. Neuroplasticity is essential for recovery from injury or disease and is also fundamental for normal development from infancy to adulthood and for learning at any age. As an example of plasticity, studies have found that children with extensive pre- or perinatal injury to left hemisphere cortical brain regions that typically serve these functions can, nonetheless, develop normally after initial delays (Levine et al., 1987; Marchman et al., 1991; Feldman et al., 1992, 2002). White matter is comprised of myelinated and unmyelinated axons that connect distant regions of the brain. A growing literature suggests that white matter contributes to neuroplasticity because white matter responds dynamically to experience (Sampaio-Baptista and Johansen-Berg, 2017). Recent studies have demonstrated that properties of white matter circuits are associated with measures of human cognition (e.g., Fink et al., 2010; Van Hecke et al., 2010; Caligiuri et al., 2015). Specifically, white matter microstructure has been linked to learning to read (Ben-shachar et al., 2007; Vandermosten et al., 2012a; Wandell and Yeatman, 2013; Travis et al., 2016a). We recently demonstrated that properties of specific white matter pathways at the onset of learning to read predicted later reading skills in a sample of children born full term (FT) (Borchers et al., 2019a). Children born preterm (PT) prior to 32 weeks gestation have been shown to have distinctive patterns of white matter microstructure as a consequence of PT birth and its complications (Volpe, 2009). In this study, in order to explore neuroplasticity in relation to white matter, we sought to determine whether similar white matter-reading associations would be found in a longitudinal study of school-aged children born PT.

White matter injury is a major component of the encephalopathy of PT preterm (PT) birth (Volpe, 2009). The immature white matter in neonates born PT is highly susceptible to injury due to hypoxia ischemia and inflammation (Back et al., 2007; Khwaja and Volpe, 2007; Volpe, 2009). These early insults affect primarily the myelin-producing cells, or oligodendrocyte precursors, and result in cell maturation arrest, cell death, and myelination failure (Back et al., 2007; Khwaja and Volpe, 2007). Even in the absence of obvious white matter injury on conventional magnetic resonance imaging (MRI), differences in the microstructure of major cerebral white matter pathways have been detected in children born extremely or very PT compared to children born near or at term, using diffusion magnetic resonance imaging (dMRI) at near term equivalent age. However, the findings have been inconsistent across studies; the PT groups have been found to have lower fractional anisotropy (FA) in some studies (Anjari et al., 2007; Knight et al., 2018), but higher FA (Giménez et al., 2008) or both higher and lower FA as a function of tract in other studies (Rose et al., 2008). Group differences on cerebral white matter pathways persist into childhood and adolescence (Nagy et al., 2003; Groeschel et al., 2014; Travis et al., 2015a) though again the results vary as a function of white matter tract and participant age. These white matter changes are likely to have implications for cognitive skills and may influence learning. Other complications of PT birth include cerebellar injury, which may also have implications for cognitive and related functioning (Brossard-Racine et al., 2015). The white matter connections between the cerebellum and cerebrum may also be altered after PT birth (Travis et al., 2016b).

We chose to study white matter microstructure and reading in children born PT for many reasons. First, reading development provides a unique opportunity to examine learning-dependent plasticity of white matter pathways in humans. Reading is typically acquired over a long period of time after instruction and many hours of practice. Second, understanding the neurobiology of reading is an educational and public health priority. Our society is becoming ever more literacy-driven. If children do not learn to read fluently, their opportunities for a fulfilling and integrated life are at risk. Third, individual differences in reading are associated with variations in white matter microstructure in samples of otherwise healthy children.

During reading, the brain integrates signals from dispersed cortical regions that process visual, phonological, and semantic information in a left-lateralized network of occipitotemporal, temporoparietal, and inferior frontal cortices (Price, 2012). In weak or impaired readers, this network of cortical regions is different, with lower levels of activation in posterior regions and greater activations in inferior frontal regions (Shaywitz et al., 2006). Functional imaging also finds, in children who respond positively to interventions, patterns of activation that begin to approach that of unimpaired readers (Simos et al., 2002; Temple et al., 2003; Gaab et al., 2007). Individual variation in the microstructural properties of several cerebral and cerebellar pathways, as measured by dMRI has been shown to correlate with reading-related skills in typically developing children and adolescents (Beaulieu et al., 2005; Ben-shachar et al., 2007; Wandell and Yeatman, 2013). White matter-reading correlations were found in the left arcuate fasciculus (Arc) and branches of the superior longitudinal fasciculus (SLF) (Vandermosten et al., 2012a; Yeatman et al., 2012a; Travis et al., 2016a). These pathways are considered to be part of the dorsal stream in cognitive models of language and reading and are thought to be involved in auditory-to-motor mapping, phonological processing, repetition, and the processing of complex sentences (Ben-shachar et al., 2007; Hickok and Poeppel, 2007; Dick and Tremblay, 2012; Skeide and Friederici, 2016). Additionally, a significant relationship between properties of the left inferior fronto-occipital fasciculus (IFOF) and performance on non-word reading suggested that this tract may be involved in highly demanding tasks translating orthography to phonology (Rollans et al., 2017). Correlations of white matter microstructure and reading were also found in the left inferior longitudinal fasciculus (ILF) (Yeatman et al., 2012a) and bilateral uncinate fasciculus (UF) (Travis et al., 2016a). The IFOF, ILF, and UF pathways are considered to be part of the ventral stream of the language and reading network and are thought to be involved in semantic and visual-orthographic processing (Friederici and Gierhan, 2013; Gil-Robles et al., 2013). The cerebellum is also known to play an important role in reading (Fiez and Petersen, 1998). White matter microstructure of the cerebellar peduncles, tracts connecting cerebellum to cerebrum, have also been implicated in reading performance (Travis et al., 2015b).

Studies have demonstrated that diffusion metrics of reading-related pathways at younger ages predict reading proficiency at older ages (Hoeft et al., 2011; Yeatman et al., 2012a; Myers et al., 2014). A recent study of children with a range of reading abilities showed that FA of the dorsal pathways, including the left Arc and the left and right SLF, and the left inferior cerebellar peduncle (ICP) at age 6 years was associated with reading outcome at age 8 years (Borchers et al., 2019a). Observations in this study were powerful because the associations to later reading persisted even after consideration of demographic covariates (sex and socioeconomic status) and individual variation in pre-literacy skills (language abilities and phonological awareness), all of which were correlated with reading outcome. Taken together, these previous longitudinal studies have established a predictive role for both dorsal and ventral stream language pathways, as well as cerebellar pathways, in predicting reading development in healthy FT children. Less is known about the predictive value of these pathways in children born PT.

Previous studies and meta-analyses confirm that children born PT are at-risk for poor reading outcome later in life (Aarnoudse-Moens et al., 2009; Kovachy et al., 2015). Studies have also suggested that white matter microstructure of tracts associated with reading or reading-related skills are different in PT and FT children. For example, in a dMRI study, the SLF was found to be associated with reading in a group of 16-year old adolescents born PT but not in peers born FT (Frye et al., 2010). A different study, also assessing 16-year old adolescents born PT and FT found that the PT group relied more on bilateral white matter tracts than did the FT group (Mullen et al., 2011). In a study of children born PT and FT across a wide age range, concurrent associations of reading and white matter metrics were found in segments of dorsal and ventral pathways (Travis et al., 2016a). However, the direction of association was different between the two birth groups suggesting that plasticity changes after PT birth lead to different neural organization in order to accomplish learning to read. In a different cohort of 6-year old children born PT and FT, pre-literacy skills were associated with microstructural properties of the left Arc (Dodson et al., 2018). Though the direction of associations was similar in the children born PT and FT, the associations were weaker in the PT group. However, associations of language and white matter properties of the right UF, a pathway within the ventral stream, were moderated by birth group status: positive associations were found in children born FT but not in children born PT (Dodson et al., 2018). These findings further support the view that variations in the underlying neurobiology of pre-literacy skills reflect plasticity and reorganization following early white matter injury, resulting in a different neural implementation of reading skills.

In this study we sought to examine whether microstructural properties of dorsal and cerebellar white matter pathways at age 6 years are associated with reading outcome at age 8 years in a sample of children born PT, as we have previously demonstrated in a sample of children born FT (Borchers et al., 2019a). We further sought to determine if the prediction is found above and beyond the contribution of demographic and pre-literacy skills at age 6 years. In addition to the dorsal and cerebellar pathways previously implicated in prediction, we included ventral stream pathways (IFOF, ILF, and UF) to determine if the microstructural properties of these pathways are associated with later reading. If the pattern of associations in PT children parallels the one seen in FT children, the findings would imply similar neural correlates of reading across birth groups, despite the fact that children born PT are at risk for white matter injuries and have previously shown different values of white matter diffusion metrics. In contrast, distinct patterns of associations in PT compared to FT children might suggest important variations in how the brain adapts to reading in children born PT.

## Materials and Methods

### Participants

Children born PT and FT were enrolled in the study at age 6 years, and followed up at age 8 years, as part of a longitudinal study that examined the neural basis of reading. The FT group (Borchers et al., 2019a) was defined as birth at ≥37 weeks gestational age or birth weight ≥2,500 g. PT birth was defined as ≤32 weeks gestational age because these are children at high risk for white matter injury (Volpe, 2009) and decrements in reading ability (Aarnoudse-Moens et al., 2009; Kovachy et al., 2015). Children were excluded from the study if they had any neurological or medical condition (other than prematurity or its complications) that might impact learning to read, including genetic disorders, significant hearing loss or visual impairment, intelligence quotient ≤ 80, and non-English speakers. The final sample included 37 FT children (15 males; mean age at time 1: 6 years 2 ± 2 month; mean age at time 2: 8 years 1 ± 2 month) and 34 PT children (22 males; mean age at time 1: 6 years 2 ± 2 month; mean age at time 2: 8 years 2 ± 2 month). To characterize the birth groups, at age 6 years, parents completed a comprehensive demographic and health questionnaire. Socioeconomic status (SES) was measured using a modified version of the Hollingshead Four Factor Index of Socioeconomic Status (Hollingshead, 1975). Children were classified as ‘bilingual’ if a parent reported that their child could speak a language other than English. All children were competent in English, attended English-speaking schools for at least two years prior to enrollment, and completed all assessments in English. Children were categorized as having a family history of reading delay if any first-degree relatives (mother, father, or siblings) were diagnosed or suspected of having a reading disorder.

### Neurocognitive Assessment at Age 6 and 8 Years

We followed the same protocol for neurocognitive assessments as described in Borchers et al. (2019a). At age 6 years, children completed standardized assessments of phonological awareness (Comprehensive Test of Phonological Processing, CTOPP; Wagner et al., 1999), language [Clinical Evaluation of Language Fundamentals – Fourth Edition (CELF-4); Semel et al., 2004], and non-verbal IQ [Weschler Abbreviated Scale of Intelligence-II (WASI-II); Wechsler and Hsiao-pin, 2011] as potential predictor variables for later reading. At age 8 years, children’s reading proficiency was assessed using the Gray Oral Reading Tests – Fifth Edition (GORT-5; Wiederholt and Bryant, 2012). The GORT requires children to read aloud stories of increasing difficulty and subsequently answer questions about the passage. The Oral Reading Index, our primary outcome variable, measures reading fluency, comprised of accuracy and rate of reading, and comprehension.

### MRI Acquisition and Analyses

Imaging parameters and methods for dMRI data preprocessing, analyses of subject’s motion, and individual native-space tractography have been described in several previous publications (Dodson et al., 2018; Borchers et al., 2019a; Bruckert et al., 2019) and are briefly summarized below.

MRI scans were obtained at age 6 years using a 3T MRI scanner (GE MR750 Discovery, GE Healthcare, Milwaukee, WI, United States) with a 32-channel head coil. High resolution T1-weighted images were collected with a 3D fast-spoiled gradient (FSPGR) sequence (TR = 7.24 ms; TE = 2.78 ms; FOV = 230 mm × 230 mm; acquisition matrix = 256 × 256; 0.9 mm isotropic voxels; orientation = sagittal). Diffusion data were collected with a dual-spin echo, echo-planar imaging sequence with full brain coverage (TR = 8300 ms; TE = 83.1 ms; FOV = 220 mm × 220 mm; acquisition matrix = 256 × 256; voxel size: 0.8594 mm × 0.8594 mm × 2 mm; orientation = axial) using a *b*-value of 1000 s/mm^2^, sampling along 30 isotropically distributed diffusion directions. Three additional volumes were acquired at *b* = 0 at the beginning of each scan.

The open-source software mrDiffusion^1^ implemented in MATLAB R2014a (Mathworks, Natick, MA, United States) was used to pre-process the diffusion data. We quantified the degree of relative head motion in each participant by calculating the magnitude of motion correction (in voxels) in the x-y-z plane of each volume relative to the prior volume. For each diffusion scan, we counted the number of volumes with translational motion of 1 voxel or more. We then calculated the mean number of volumes with ≥1 voxel of relative motion across the entire sample (*M* = 1.37, *SD* = 3.18). Participants who deviated from this mean by more than three standard deviations were excluded from analyses. This procedure led to the exclusion of two participants (1 PT). Group comparisons were performed to ensure that children born FT and PT did not differ in their average relative head motion [*t*(69) = 1.58, *p* = 0.118, *d* = 0.36].

In the remaining participants, to correct for participant’s motion, each diffusion weighted image was registered to the mean of the three non-diffusion (b0) images using a rigid body transformation (Rohde et al., 2004). The mean b0 image was registered to the participant’s T1-weighted image, which had been aligned to the canonical ac-pc orientation. The combined transform that resulted from motion correction and alignment to the T1 anatomy was applied to the raw data once, and the transformed images were resampled to 2 mm × 2 mm × 2 mm isotropic voxels. Following robust tensor fitting and outlier rejection based on the RESTORE procedure (Chang et al., 2005), FA maps were generated using the standard formula (Mukherjee et al., 2008).

Cerebral and cerebellar white matter pathways were tracked and segmented using the open source software Automated Fiber Quantification (AFQ; Yeatman et al., 2012b). AFQ uses a three-step procedure to identify white matter pathways in the native space of each child: (i) Whole-brain tractography was performed using a deterministic streamline tracking algorithm (Mori et al., 1999; Chang et al., 2005), with a fourth-order Runge–Kutta path integration method. Tractography was seeded from each voxel in a white matter mask (FA > 0.2). Tracking proceeded in all directions until FA values dropped below 0.15, or until the angle between the last path segment and next step direction was greater than 30°; (ii) Automatic tract segmentation was done using a way-point region of interest (ROIs) approach as described by Wakana et al. (2007). Template ROIs were defined in MNI space and warped to native space by applying a non-linear transformation (Friston and Ashburner, 2004); and (iii) Automatic tract refinement was achieved by comparing each candidate fiber to an established fiber tract probability map (Hua et al., 2008) and removing streamlines that pass through regions of white matter having a low probability for belonging to the tract under analysis.

**FIGURE 1:**
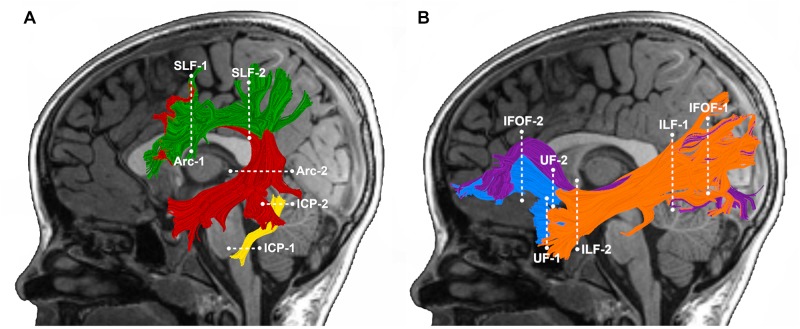
Tractography of cerebral and cerebellar white matter pathways. Left hemisphere tract renderings are displayed on a mid-sagittal T1 image from a representative child in our group. Right hemisphere tract renderings not shown. Dashed lines represent the location of the regions of interest (ROIs) used to segment each pathway from the whole-brain tractogram. **(A)** Dorsal stream and cerebellar pathways: the left arcuate fasciculus (Arc) is displayed in *red*, the left superior longitudinal fasciculus (SLF) in *green*, and the left inferior cerebellar peduncle (ICP) in *yellow*. **(B)** Ventral stream pathways: the left inferior frontal occipital fasciculus (IFOF) is displayed in purple, inferior longitudinal fasciculus (ILF) in *orange* and the left uncinate fasciculus (UF) in *blue*.

We selected pathways for analysis *a priori* based on our previous findings, documenting associations between the mean tract-FA of these pathways at age 6 years and reading outcome at age 8 years in the subsample of children born FT (Borchers et al., 2019a) (Figure 1A). These pathways included: left Arc, left and right SLF, and left ICP. We also included the left and right IFOF, ILF, and UF (Figure 1B) to examine whether ventral stream pathways are associated with later reading in children born PT. Individual tractograms (fiber renderings) of each pathway were visually inspected in each child prior to any statistical analysis, to ensure that the tract generally conformed to anatomical norms for location and shape and did not include many aberrant fibers. We successfully identified the tracts in all children with the following exceptions: the left AF could not be tracked in one FT child and the left ICP could not be tracked in one FT and three PT children.

### Statistical Analyses

Statistical analyses were conducted using IBM SPSS software (version 23.0, IBM Corp., 2015). Statistical significance was set at *p* < 0.05. The Shapiro–Wilk test was used to assess whether our neurocognitive and neurobiological data were normally distributed. With the exception of SES and mean tract-FA of the left Arc, all data were normally distributed. We chose to do parametric tests for all associations. Bonferroni correction was applied to these zero-order associations to account for multiple comparisons.

#### Demographic Characteristics of Children Born PT and FT

We compared children born PT and FT on four continuous (age, gestational age, birthweight, SES) and four categorical (sex, language status, grade, family history of reading delay) demographic measures using two-tailed independent *t*-tests or chi-square analyses, respectively. We further compared the two groups on their standardized measures of non-verbal IQ, language, and phonological awareness as well as mean tract-FA obtained at age 6 years and reading outcome at age 8 years using two-tailed independent *t*-tests. Two-tailed independent *t*-tests were also used to evaluate possible confounding effects at age 6 years with reading outcome at age 8 years by comparing the Oral Reading Index in the following subgroups: male vs. female, monolingual vs. bilingual. The Mann–Whitney *U*-test was used to compare reading outcome between subgroups with uneven distributions: kindergarten vs. first grade and positive vs. negative family history of reading delay. We computed Pearson correlations to assess the degree of associations between reading outcome at age 8 years and each of the following at age 6 years: SES, non-verbal IQ, language, and phonological awareness.

#### Hierarchical Linear Regression Models

We conducted a series of hierarchical linear regression models to assess the contribution of mean tract-FA of the selected tracts at age 6 years to reading outcome at age 8 years. Demographic variables and neurocognitive measures that showed significant subgroup differences or associations with reading outcome were included as covariates in all models. We first measured whether there was a main effect of each white matter pathway and then whether birth group status moderated the prediction of mean tract-FA to later reading outcome. We repeated the series of regression models to assess the unique contribution of each white matter pathway after consideration of language and phonological awareness – two pre-literacy skills that were previously associated with reading outcome in FT children (Borchers et al., 2019a). The variance inflation factor (VIF) was calculated to assess multicollinearity of each model. We considered VIF values less than 10 to indicate that there was no concern for multicollinearity (Field, 2013).

## Results

### Characteristics of PT and FT Children

Group characteristics and statistical comparisons between PT and FT children are summarized in Table 1. By design, children born PT had significantly lower gestational age and birthweight than their FT peers. Overall, the two groups were well-matched on age, ethnicity, language status, and grade. The PT group had significantly more boys, but significantly fewer children with a family history of reading delay compared to the FT group. While children born PT and FT were on average from high SES backgrounds, SES was significantly lower in the PT group. Within the PT group, four had intrauterine growth restriction, 29 children had respiratory distress syndrome, of which five were classified as severe and required mechanical ventilation; six developed evidence of chronic lung disease, requiring supplemental oxygen beyond 36 weeks gestation. A total of 27 children had hyperbilirubinemia requiring phototherapy. Twelve children had abnormalities on cranial ultrasound; nine had intraventricular hemorrhage Grade II or less, two had mild dilatation of the ventricles, one had white matter injury and one had parenchymal injury. A total of 18 children had near term MRI scans and seven had abnormal findings, of which five had evidence of white matter changes or enlarged ventricles. However, no child had frank cystic lesions.

**Table 1:** Characteristics of the sample (significant *p*-values are printed in bold).

*N* = 71	Full Term (*n* = 37) *M* (*SD*) or *n* (%)	Preterm (*n* = 34) *M* (*SD*) or *n* (%)	*t* or χ^2^	*p*	*d*
**Demographic measures**				
Age of test 1	6.2 (0.2)	6.1 (0.2)	0.60	0.548	0.12
Gestational age at birth (wks)	39.5 (1.5)	29.5 (2.4)	21.16	**<0.001**	4.98
Birth weight (grams)	3,298 (399)	1,336 (468)	19.05	**<0.001**	4.51
Males # (%)	15 (41%)	22 (69%)	4.15	**0.042**	n/a
Bilingual # (%)	19 (51%)	11 (32%)	2.62	0.105	n/a
Kindergarten # (%)	25 (68%)	26 (76%)	0.69	0.405	n/a
FH of reading delay # (%)	8 (22%)	1 (3%)	5.59	**0.018**	n/a
SES^1^	58.2 (10.0)	52.3 (14.2)	2.01	**0.049**	0.48
**Neurocognitive measures**				
IQ^2^	112.3 (16.0)	101.3 (13.4)	3.12	**0.003**	0.74
Language^3^	113.2 (12.8)	102.5 (10.8)	3.79	**<0.001**	0.90
Phono awareness^4^	113.5 (11.8)	110.1 (13.0)	1.15	0.255	0.27
Reading outcome^5^	102.2 (13.2)	97.6 (10.9)	1.61	0.112	0.38
**Mean tract-fractional anisotropy**				
Arc-L^6^	0.49 (0.03)	0.47 (0.03)	1.98	0.052	0.67
SLF-L^7^	0.42 (0.04)	0.43 (0.04)	-0.73	0.467	0.25
SLF-R^7^	0.47 (0.05)	0.48 (0.04)	-0.64	0.525	0.22
ICP-L^8^	0.43 (0.04)	0.44 (0.04)	-1.26	0.212	0.25
IFOF-L^9^	0.49 (0.03)	0.50 (0.04)	-0.76	0.445	0.18
IFOF-R^9^	0.50 (0.03)	0.50 (0.03)	-1.18	0.240	0.29
ILF-L^10^	0.43 (0.02)	0.43 (0.03)	0.40	0.688	0.10
ILF-R^10^	0.43 (0.02)	0.43 (0.03)	-0.54	0.589	0.13
UF-L^11^	0.43 (0.02)	0.42 (0.04)	0.85	0.401	0.20
UF-R^11^	0.45 (0.02)	0.44 (0.03)	1.57	0.123	0.38

*^1^Socioeconomic status, measured with the Hollingshead Index at age 6 years; ^2^Non-verbal Intelligence Quotient, measured with the WASI-II at age 6 years; ^3^Core Language Index, measured with the CELF-4 at age 6 years; ^4^Phonological Awareness Composite, measured with the CTOPP at age 6 years; ^5^Oral Reading Index, measured with the GORT-5 at age 8 years; ^6^Arcuate fasciculus; ^7^Superior longitudinal fasciculus; ^8^Inferior fronto-occipital fasciculus; ^9^Inferior cerebellar peduncle; ^10^Inferior longitudinal fasciculus; ^11^Unciate fasciculus. L, left; R, right.*

Children born PT and FT scored within the normal range on all cognitive, pre-literacy, and reading assessments (Table 1). While the two groups did not significantly differ in phonological awareness at age 6 years or reading outcome at age 8 years, PT children had significantly lower mean scores on language and non-verbal IQ compared to their FT peers. We found no significant differences in mean tract-FA of any white matter pathway.

To determine whether demographic variables or neurocognitive measures at age 6 years were important contributors to reading at age 8 years, we examined subgroup differences in, and correlations with reading outcome (Supplementary Tables S1, S2). Across children born PT and FT, girls scored significantly higher than boys on reading outcome (Supplementary Table S1). Therefore, sex was entered as a covariate in all regression models. Reading outcome did not differ between children who were monolingual vs. bilingual, between children enrolled in kindergarten vs. first grade at age 6 years, or between children with vs. without a family history of reading delay. Thus, we did not covary for these variables in subsequent regression models despite the difference in proportion of children with a family history of reading delay in each birth group. Across the entire sample, reading outcome at age 8 years was significantly correlated with SES, non-verbal IQ, language, and phonological awareness skills at age 6 years (Supplementary Table S2).

Based on these preliminary subgroup and correlation analyses, sex, SES, and non-verbal IQ were consistently entered as covariates in all regression models. Language and phonological awareness were added as behavioral predictor variables in the second round of regression models. Correlations between reading outcome and demographic variables, pre-literacy skills, and mean tract-FA ranged from -0.12 to 0.61 (Supplementary Tables S2, S3). For all subsequent regression models the VIF values were equal or less than 4.0. Thus, there was no cause for concern regarding multicollinearity.

### Associations Between White Matter Pathways and Reading Outcome

**FIGURE 2:**
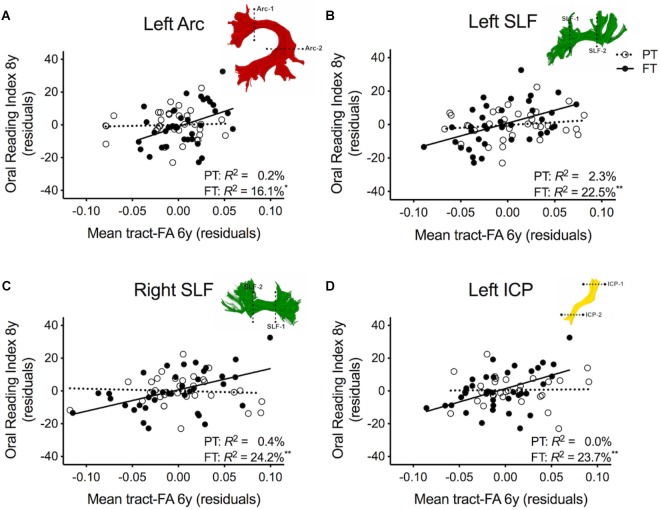
Regression residuals of mean tract-FA of the dorsal stream and cerebellar pathways at age 6 years and reading outcome at age 8 years in children born preterm (PT, open circle, dotted line) and full term (FT, filled circles, solid line) after controlling for sex, socioeconomic status, and non-verbal IQ. Subplots **(A–D)** show a significant interaction indicating that associations between reading outcome and mean tract-FA of the left Arcuate (Arc), left and right superior longitudinal fasciculus (SLF), and left inferior cerebellar peduncle (ICP) differed between birth groups. Δ *R*^2^ values reflect the increased variance accounted for by the corresponding pathway in children born PT or FT.

Table 2 shows the results of multiple regression models predicting reading outcome at age 8 years by mean tract-FA of the left Arc, left and right SLF, and left ICP at age 6 years controlling for sex, SES, and non-verbal IQ. *R*^2^ change and adjusted *R*^2^ change values in models 1B-I reflect the increase in explained variance associated with the addition of the main effect of tract or the interaction term of tract × birth group relative to the preceding model with tract only. *Models 1A-1I* demonstrated that non-verbal IQ at age 6 years was the only consistent covariate that explained significant unique variance in reading outcome. When controlling for all covariates, birth group was not a significant predictor variable of reading outcome (*Model 1A*). The entire model accounted for 23.3% of the variance in reading outcome. FA of the left Arc did not significantly contribute to reading outcome (*Model 1B*). However, when we entered the interaction between mean tract-FA of the left Arc and birth group in *Model 1C* we found a significant increase of 5.4% in variance accounted for. As illustrated in Figure 2A, the prediction of the left Arc to reading outcome was different in the two groups: in children born FT, mean tract-FA was positively associated with reading outcome, while mean tract-FA was not associated with reading outcome in children born PT. *Models 1C-1I* demonstrated a similar pattern of associations. In all three cases, FA of the tract made a significant contribution to the variance in reading outcome, adding 6.3–8.7% to the variance accounted for. However, the interaction of mean tract-FA with birth group was significant (*Models 1G and 1I*) or trending toward significance (*Model 1E, p* = 0.053) in all remaining cases, explaining approximately 3.9–7.8% additional variance. The associations between reading outcome at age 8 years and mean tract-FA of the left SLF, right SLF, and left ICP at age 6 years was again positive in the FT, but absent in the PT group (Figure 2B–D).

**Table 2:** Prediction of reading outcome at age 8 years by mean tract-FA of the left Arcuate (Arc-L), left and right superior longitudinal fasciculus (SLF-L, SLF-R) and left inferior cerebellar peduncle (ICP-L) at age 6 years, controlling for sex, socioeconomic status (SES), and non-verbal intelligence (IQ) in the preterm and full term groups.

	Model 1A	Model 1B	Model 1C	Model 1D	Model 1E	Model 1F	Model 1G	Model 1H	Model 1I
Sex	4.1 (2.8)	4.1 (2.8)	4.2 (2.7)	2.9 (2.7)	2.7 (2.6)	3.8 (2.7)	4.6 (2.6)	4.1 (2.7)	3.3 (2.6)
SES	0.2 (0.1)	0.1 (0.1)	0.1 (0.1)	0.2 (0.1)	0.2 (0.1)	0.1 (0.1)	0.1 (0.1)	0.1 (0.1)	0.1 (0.1)
IQ	**0.2 (0.1)^a^**	**0.3 (0.1)^a^**	**0.3 (0.1)^a^**	**0.2 (0.1)^a^**	**0.2 (0.1)^a^**	**0.2 (0.1)^a^**	**0.2 (0.1)^a^**	**0.3 (0.1)^b^**	**0.3 (0.1)^b^**
Group	-0.1 (2.8)	1.1 (2.9)	**94.8 (41.8)^a^**	-1.4 (2.7)	50.7 (26.5)	-0.9 (2.8)	**72.6 (26.1)^b^**	-0.7 (2.8)	**78.6 (28.7)^b^**
Arc-L	–	87.2 (44.7)	**198.2 (65.7)^b^**	–	–	–	–	–	–
Arc-L × group	–	–	-**193.8 (86.3)^a^**	–	–	–	–	–	-
SLF-L	–	–	–	**92.9 (32.2)^b^**	**154.8 (44.5)^c^**	–	–	–	–
SLF-L × group	–	–	–	–	-122.2 (61.9)	–	–	–	–
SLF-R	–	–	–	–	–	**69.7 (29.0)^a^**	**134.0 (35.7)^c^**	–	–
SLF-R × group	–	–	–	–	–	**-**	-**154.1 (54.5)^b^**	–	–
ICP-L	–	–	–	–	–	–	–	**84.3 (35.2)^a^**	**170.2 (45.5)^c^**
ICP-L × group	–	–	–	–	–	–	–	**-**	-**181.4 (65.3)^b^**
Δ *R*^2^	–	4.3%	5.4%^a^	8.7%^b^	3.9%	**6.3%^a^**	**7.8%^b^**	**6.3%^a^**	**7.6%^b^**
Total *R*^2^	**23.3%^c^**	**27.2%^c^**	**32.6%^c^**	**32.0%^c^**	**35.9%^c^**	**29.6%^c^**	**37.4%^c^**	**33.2%^c^**	**40.8%^c^**
Adjusted *R*^2^	**18.7%^c^**	**21.5%^c^**	**26.2%^c^**	**26.8%^c^**	**29.9%^c^**	**24.2%^c^**	**31.5%^c^**	**27.7%^c^**	**34.8%^c^**

*Data are unstandardized coefficients (SE). ^a^p < 0.05, ^b^p < 0.01, ^c^p < 0.001. R^2^ change (**Δ** R^2^) values in models 1B, D, F, and H are in reference to model 1A. **Δ** R^2^ values in models 1C, E, G, and I reflect the increase in variance accounted for by the interaction term in relation to the preceding model with the main effect for that tract. Significant values are printed in bold.*

**FIGURE 3:**
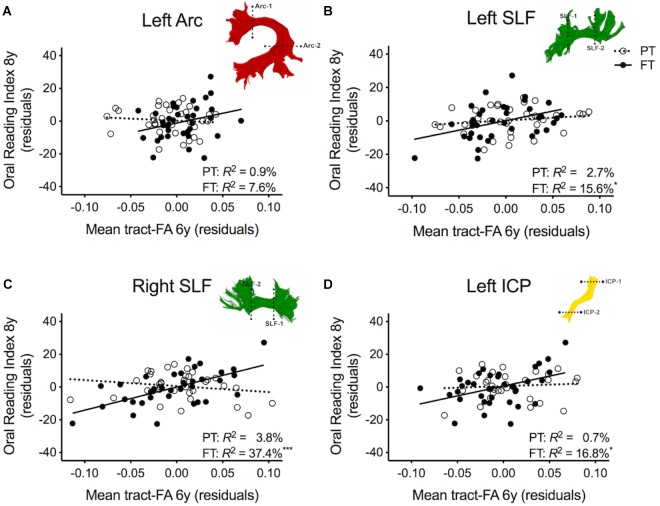
Regression residuals of mean tract-FA of the dorsal stream and cerebellar pathways at age 6 years and reading outcome at age 8 years in children born preterm (PT, open circle, dotted line) and FT (filled circles, solid line) after controlling for sex, socioeconomic status, non-verbal IQ, language, and phonological awareness. Subplots **(A,C,D)** show a significant interaction indicating that associations between reading outcome and mean tract-FA of the left Arcuate (Arc), right SLF, and left ICP differed between birth groups. Subplot **(B)** shows that the association between reading outcome and mean tract-FA of the left SLF was not significantly different between birth groups. Δ *R*^2^ values reflect the increased variance accounted for by the corresponding pathway in children born PT or FT.

Table 3 shows the second round of regression analyses, the results of adding pre-literacy skills to the models. *Model 2A* showed that the combined contribution of covariates, language, and phonological awareness improved the prediction of reading outcome, accounting for 42.4% of the variance. With the addition of pre-literacy skills to the model, non-verbal IQ was no longer a unique predictor of reading outcome. However, language and phonological awareness were significant predictors. Similar to *Model 1A*, birth group status did not explain significant variance in reading outcome at age 8 years, even after the addition of pre-literacy skills at age 6 years to the model. Overall, the pattern of results was similar with pre-literacy skills included in the models as in the first round of analyses. The interaction terms of mean tract-FA of the left Arc, left and right SLF, and left ICP remained significant and added 3.9–10.3% unique variance (*Model 2C, 2G*, and *2I*). The exception was *Model 2E*, in which the interaction between the left SLF and birth group was no longer approaching significance. However, we found a significant main effect of the left SLF accounting for 4.7% of the variance in reading outcome across both birth groups (*p* = 0.021) (*Model 2E*). In children born PT and FT, mean tract-FA of the left SLF at age 6 years was positively associated with reading outcome at age 8 years (Figure 3B). In the remaining dorsal tracts, associations were positive in the FT and non-significant in the PT groups (Figure 3C,D).

**Table 3:** Prediction of reading outcome at age 8 years by mean tract-FA of the left Arcuate (Arc-L), left and right superior longitudinal fasciculus (SLF-L, SLF-R) and left inferior cerebellar peduncle (ICP-L) at age 6 years, controlling for sex, socioeconomic status (SES), non-verbal intelligence (IQ), language, and phonological awareness (Phono aware) in the preterm and full term groups.

	Model 2A	Model 2B	Model 2C	Model 2D	Model 2E	Model 2F	Model 2G	Model 2H	Model 2I
Sex	0.9 (2.5)	1.1 (2.6)	1.3 (2.5)	0.3 (2.5)	0.2 (2.4)	0.6 (2.5)	1.3 (2.2)	1.5 (2.6)	1.2 (2.5)
SES	0.1 (0.1)	0.0 (0.1)	0.0 (0.1)	0.1 (0.1)	0.1 (0.1)	0.0 (0.1)	0.0 (0.1)	0.1 (0.1)	0.0 (0.1)
IQ	0.0 (0.1)	0.1 (0.1)	0.1 (0.1)	0.0 (0.1)	0.0 (0.1)	0.1 (0.1)	0.0 (0.1)	0.1 (0.1)	0.1 (0.1)
Language	**0.4 (0.1)^a^**	**0.4 (0.1)^a^**	**0.3 (0.1)^a^**	**0.3 (0.1)^a^**	**0.3 (0.1)^a^**	**0.4 (0.1)^b^**	**0.5 (0.1)^c^**	0.2 (0.2)	0.3 (0.1)
Phono aware	**0.3 (0.1)^a^**	0.2 (0.1)	0.2 (0.1)	**0.2 (0.1)^a^**	0.2 (0.1)	**0.2 (0.1)^a^**	**0.2 (0.1)^a^**	**0.3 (0.1)^a^**	0.2 (0.1)
Group	1.1 (2.6)	1.7 (2.8)	**81.3 (38.0)^a^**	0.2 (2.5)	37.1 (24.3)	0.6 (2.5)	86.5 (22.2)^c^	0.0 (2.7)	**60.1 (27.9)^a^**
Arc-L	–	35.8 (43.6)	**132.5 (62.6)^a^**	–	–	–	–	–	–
Arc-L × group	–	–	**-164.7 (78.4)^a^**	–	–	–	–	–	–
SLF-L	–	–	–	**69.9 (29.4)^a^**	**115.2 (41.6)^b^**	–	–	–	–
SLF-L × group	–	–	–	–	**-**86.6 (56.8)	–	–	–	–
SLF-R	–	–	–	–	–	**62.4 (25.8)^a^**	**138.2 (30.3)^c^**	**–**	**–**
SLF-R × group	–	–	–	–	–	**-**	**-179.2 (46.0)^c^**	**-**	**-**
ICP-L	–	–	–	–	–	**-**	**-**	**72.1 (33.0)^a^**	**136.6 (43.7)^b^**
ICP-L × group	–	–	–	–	–	**–**	**–**	**–**	**-137.3 (63.3)^a^**
Δ *R*^2^	–	0.6%	**3.9%^a^**	4.7%^a^	1.9%	**4.9%^a^**	**10.3%^c^**	**4.4%^a^**	**4.0%^a^**
Total *R*^2^	**42.4%^c^**	**42.7%^c^**	**46.6%^c^**	**47.2%^c^**	**49.1%^c^**	**47.3%^c^**	**57.7%^c^**	**46.1%^c^**	**50.1%^c^**
Adjusted *R*^2^	**37.0%^c^**	**36.2%^c^**	**39.6%^c^**	**41.3%^c^**	**42.5%^c^**	**41.5%^c^**	**52.2%^c^**	**39.7%^c^**	**43.2%^c^**

*Data are unstandardized coefficients (SE). ^a^p < 0.05, ^b^p < 0.01, ^c^p < 0.001. R^2^ change (**Δ** R^2^) values in models 2B, D, F, and H are in reference to model 2A. **Δ** R^2^ values in models 2C, E, G, and I reflect the increase in variance accounted for by the interaction term in relation to the preceding model with the main effect for that tract. Significant values are printed in bold.*

Since PT and FT children differed in their proportion of children with a family history of reading delay, regression models might have been unduly influenced by these children. We therefore, re-ran the regression models excluding children with a family history of reading delay (*n* = 9; 1 PT). The pattern of associations between the selected white matter pathways at age 6 years and reading outcome at age 8 years remained the same – before and after consideration of pre-literacy skills (Supplementary Tables S4, S5).

**FIGURE 4:**
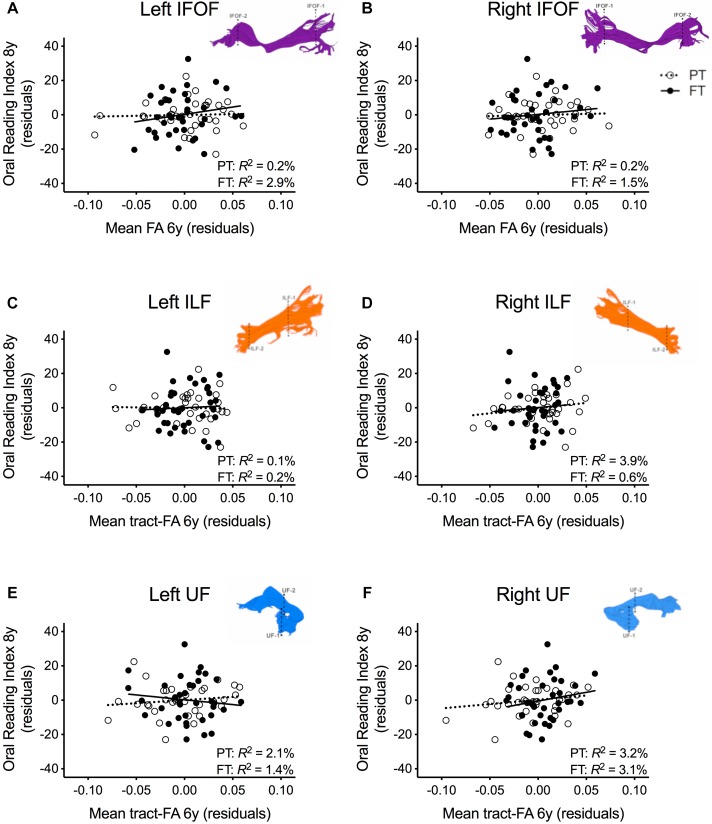
Regression residuals of mean tract-FA of the ventral stream pathways at age 6 years and reading outcome at age 8 years in children born preterm (PT, open circle, dotted line) and FT (filled circles, solid line) after controlling for sex, socioeconomic status, and non-verbal IQ. Subplots **(A–F)** show no significant associations between reading outcome and mean tract-FA of the left and right inferior frontal occipital fasciculus (IFOF), ILF, and UF. Δ *R*^2^ values reflect the increased variance accounted for by the corresponding pathway in children born PT or FT.

**FIGURE 5:**
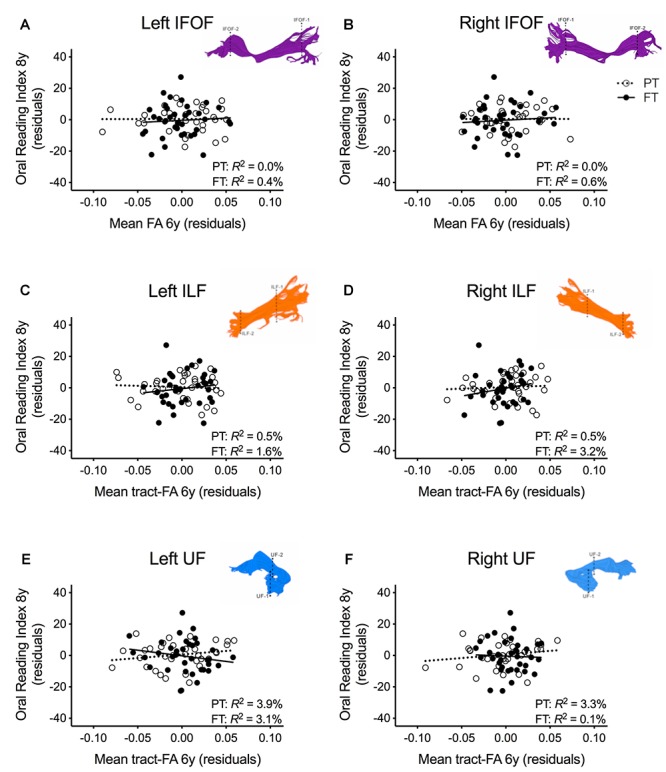
Regression residuals of mean tract-FA of the ventral stream pathways at age 6 years and reading outcome at age 8 years in children born preterm (PT, open circle, dotted line) and FT (filled circles, solid line) after controlling for sex, socioeconomic status, non-verbal IQ, language, and phonological awareness. Subplots **(A–F)** show no significant associations between reading outcome and mean tract-FA of the left and right inferior frontal occipital fasciculus (IFOF), ILF, UF. Δ *R*^2^ values reflect the increased variance accounted for by the corresponding pathway in children born PT or FT.

In order to assure that we did not miss any potential associations between mean tract-FA of other reading-related pathways and reading outcome in the PT group, we conducted a final round of regression analyses including the left and right IFOF, ILF, and UF. We did not find any main effects of these ventral pathways controlling for sex, SES, and non-verbal IQ at age 6 years or any significant interactions tract with birth group (Table 4, Figure 4A–F). After pre-literacy skills were added to the models, the interaction of mean tract-FA of the left UF and birth group (Model *4K*) approached significance (*p* = 0.055) explaining 3.5% of the variance in reading outcome (Table 5). Neither the interaction with birth group nor the main effect of the left or right IFOF, left or right ILF, or left or right UF added significant variance to the model after consideration of pre-literacy skills (Table 5, Figure 5A–F).

**Table 4:** Prediction of reading outcome at age 8 years by mean tract-FA of the left and right inferior frontal-occipital fasciculus (IFOF-L, IFOF-R), inferior longitudinal fasciculus (ILF-L, ILF-R), and uncinate fasciculus (UF-L, UF-R) at age 6 years, controlling for sex, socioeconomic status (SES), and non-verbal intelligence (IQ) in the preterm and full term groups.

	Model 3A	Model 3B	Model 3C	Model 3D	Model 3E	Model 3F	Model 3G	Model 3H	Model 3I	Model 3J	Model 3K	Model 3L	Model 3M
Sex	4.1 (2.8)	4.6 (2.8)	4.6 (2.8)	4.1 (2.8)	3.7 (2.9)	4.1 (2.8)	4.1 (2.8)	3.7 (2.8)	3.7 (2.8)	4.2 (2.9)	5.3 (2.9)	4.9 (2.8)	4.7 (2.9)
SES	0.2 (0.1)	0.2 (0.1)	0.2 (0.1)	0.2 (0.1)	0.2 (0.1)	0.2 (0.1)	0.2 (0.1)	0.2 (0.1)	0.2 (0.1)	0.2 (0.1)	0.2 (0.1)	0.2 (0.1)	0.2 (0.1)
IQ	**0.2 (0.1)^a^**	**0.2 (0.1)^a^**	**0.2 (0.1)^a^**	**0.2 (0.1)^a^**	**0.2 (0.1)^a^**	**0.2 (0.1)**	**0.2 (0.1)^a^**	**0.2 (0.1)^a^**	**0.2 (0.1)^a^**	**0.2 (0.1)^a^**	**0.2 (0.1)^a^**	**0.2 (0.1)^a^**	**0.2 (0.1)^a^**
Group	**-**0.1 (2.8)	**-**0.4 (2.9)	24.8 (44.5)	**-**0.4 (2.9)	29.8 (49.3)	**–0.1 (2.9)^a^**	37.2 (42.1)	**-**0.4 (2.9)	20.7 (52.4)	**-**0.1 (2.9)	**-**63.2 (39.9)	0.8 (2.9)	14.5 (49.4)
IFOF-L	–	36.7 (44.5)	69.7 (73.5)	–	–	–	–	–	–	–	–	–	–
IFOF-L × group	–	–	-51.0 (90.0)	–	–	–	–	–	–	–	–	–	–
IFOF-R	–	–	–	33.7 (47.9)	65.2 (70.3)	–	–	–	–	–	–	–	–
IFOF-R × group	–	–	–	–	-60.7 (98.8)	–	–	–	–	–	–	–	–
ILF-L	–	–	–	–	–	4.4 (48.5)	61.6 (80.7)	–	–	–	–	–	–
ILF-L × group	–	–	–	–	–	–	-86.9 (97.8)	–	–	–	–	–	–
ILF-R	–	–	–	–	–	–	–	58.0 (56.4)	91.4 (100.6)	–	–	–	–
ILF-R × group	–	–	–	–	–	–	–	–	-49.1 (121.8)	–	–	–	–
UF-L	–	–	–	–	–	–	–	–	–	1.4 (44.3)	-93.5 (74.2)	–	–
UF-L × group	–	–	–	–	–	–	–	–	–	–	148.5 (93.6)	–	–
UF-R	–	–	–	–	–	–	–	–	–	–	–	67.8 (49.7)	88.7 (90.5)
UF-R × group	–	–	–	–	–	–	–	–	–	–	–	–	-30.8 (111.2)
Δ *R*^2^	–	0.8%	0.4%	0.6%	0.4%	0.0%	0.9%	1.2%	0.2%	0.0%	2.9%	2.1%	0.1%
Total *R*^2^	**23.3%^c^**	**24.1%^b^**	**24.5%^b^**	**23.9%^b^**	**24.3%^b^**	**23.3%^b^**	**24.3%^b^**	**24.5%^b^**	**24.7%^b^**	**23.3%^b^**	**26.2%^b^**	**25.5%^b^**	**25.5%^b^**
Adjusted *R*^2^	**18.7%^c^**	**18.3%^b^**	**17.4%^b^**	**18.0%^b^**	**17.3%^b^**	**17.4%^b^**	**17.2%^b^**	**18.7%^b^**	**17.7%^b^**	**17.4%^b^**	**19.3%^b^**	**19.7%^b^**	**18.6%^b^**

*Data are unstandardized coefficients (SE). ^a^p < 0.05, ^b^p < 0.01, ^c^p < 0.001. R^2^ change (**Δ** R^2^) values in models 3B, D, F, H, J, and L are in reference to model 3A. R^2^ change values in models 3C, E, G, I, K, and M reflect the increase in variance accounted for by the interaction term in relation to the preceding model with the main effect for that tract. Significant values are printed in bold.*

**Table 5:** Prediction of reading outcome at age 8 years by mean tract-FA of the left and right inferior frontal-occipital fasciculus (IFOF-L, IFOF-R), inferior longitudinal fasciculus (ILF-L, ILF-R), and uncinate fasciculus (UF-L, UF-R) at age 6 years, controlling for sex, socioeconomic status (SES), non-verbal intelligence (IQ), language, and phonological awareness (Phono aware) in the preterm and full term groups.

	Model 4A	Model 4B	Model 4C	Model 4D	Model 4E	Model 4F	Model 4G	Model 4H	Model 4I	Model 4J	Model 4K	Model 4L	Model 4M
Sex	0.9 (2.5)	1.08 (2.6)	1.1 (2.6)	1.0 (2.6)	0.7 (2.6)	1.0 (2.6)	0.9 (2.6)	0.7 (2.6)	0.4 (2.6)	1.0 (2.6)	2.1 (2.6)	1.3 (2.6)	1.6 (2.7)
SES	0.1 (0.1)	0.1 (0.1)	0.1 (0.1)	0.1 (0.1)	0.1 (0.1)	0.1 (0.1)	0.0 (0.1)	0.1 (0.1)	0.0 (0.1)	0.1 (0.1)	0.0 (0.1)	0.1 (0.1)	0.1 (0.1)
IQ	0.0 (0.1)	0.0 (0.1)	0.0 (0.1)	0.0 (0.1)	0.1 (0.1)	0.0 (0.1)	0.1 (0.1)	0.1 (0.1)	0.1 (0.1)	0.0 (0.1)	0.0 (0.1)	0.0 (0.1)	0.0 (0.1)
Language	**0.4 (0.1)^a^**	**0.4 (0.1)^a^**	**0.4 (0.2)^a^**	**0.4 (0.1)**	**0.3 (0.2)^a^**	**0.4 (0.1)^a^**	**0.4 (0.1)^a^**	**0.3 (0.1)^a^**	**0.4 (0.1)^a^**	**0.4 (0.1)^a^**	**0.4 (0.1)^a^**	**0.3 (0.2)^a^**	**0.4 (0.2)^a^**
Phono aware	**0.3 (0.1)^a^**	**0.3 (0.1)^a^**	**0.3 (0.1)^a^**	0.3 (0.1)	**0.3 (0.1)^a^**	**0.3 (0.1)^a^**	**0.3 (0.1)^a^**	**0.3 (0.1)^a^**	**0.3 (0.1)^a^**	**0.3 (0.1)^a^**	**0.3 (0.1)^a^**	**0.3 (0.1)^a^**	**0.3 (0.1)^a^**
Group	1.1 (2.6)	1.1 (2.6)	7.6 (40.0)	1.0 (2.7)	24.0 (44.3)	1.2 (2.6)	46.3 (36.9)	0.9 (2.6)	61.8 (46.6)	1.2 (2.6)	-68.0 (34.8)	1.4 (2.7)	-25.3 (45.1)
IFOF-L	–	10.0 (39.9)	18.7 (66.3)	–	–	–	–	–	–	–	–	–	–
IFOF-L × group	–	–	-13.2 (81.0)	–	–	–	–	–	–	–	–	–	–
IFOF-R	–	–	–	14.1 (42.6)	38.5 (63.5)	–	–	–	–	–	–	–	–
IFOF-R × group		–	–	–	-46.3 (88.9)	–	–	–	–	–	–	–	–
ILF-L	–	–	–	–	–	8.3 (42.7)	77.6 (70.8)	–	–	–	–	–	–
ILF-L × group	–	–	–	–	–	–	-105.2 (85.9)	–	–	–	–	–	–
ILF-R	–	–	–	–	–	–	–	45.1 (49.9)	140.7 (88.2)	–	–	–	–
ILF-R × group	–	–	–	–	–	–	–	–	-141.5 (108.0)	–	–	–	–
UF-L	–	–	–	–	–	–	–	–	–	2.3 (39.0)	-102.3 (64.9)	–	–
UF-L × group	–	–	–	–	–	–	–	–	–	–	163.1 (81.9)	–	–
UF-R	–	–	–	–	–	–	–	–	–	–	–	27.6 (45.7)	-15.1 (85.3)
UF-R × group	–	–	–	–	–	–	–	–	–	–	–	–	60.4 (101.7)
Δ *R*^2^	–	0.1%	0.0%	0.1%	0.3%	0.0%	1.4%	0.7%	1.5%	0.0%	3.5%	0.3%	0.3%
Total *R*^2^	**42.4%^c^**	**42.5%^c^**	**42.5%^c^**	**42.5%^c^**	**42.8%^c^**	**42.5%^c^**	**43.8%^c^**	**43.2%^c^**	**44.7%^c^**	**42.4%^c^**	**45.9%^c^**	**42.8%^c^**	**43.1%^c^**
Adjusted *R*^2^	**37.0%^c^**	**36.1%^c^**	**35.1%^c^**	**36.2%^c^**	**35.4%^c^**	**36.1%^c^**	**36.6%^c^**	**36.9%^c^**	**37.6%^c^**	**36.1%^c^**	**38.9%^c^**	**36.4%^c^**	**35.8%^c^**

*Data are unstandardized coefficients (SE). ^a^p < 0.05, ^b^p < 0.01, ^c^p < 0.001. R^2^ change (**Δ** R^2^) values in models 4B, D, F, H, J, and L are in reference to model 4A. R^2^ change values in models 4C, E, G, I, K, and M reflect the increase in variance accounted for by the interaction term in relation to the preceding model with the main effect for that tract. Significant values are printed in bold.*

## Discussion

This study demonstrated that brain-reading relations were different in children born PT and FT. Individual differences in white matter properties of the left Arc, left and right SLF, and left ICP at age 6 years were associated with reading outcome at age 8 years in children born FT (Borchers et al., 2019a). Despite comparable levels (between birth groups) of mean tract-FA of these four selected pathways and similar reading scores, we found that birth group moderated the associations between FA at age 6 years and reading outcome 2 years later. Mean tract-FA of these pathways was not associated with reading outcome in children born PT. The pattern of results did not change after excluding nine children with a family history of reading delay. We did not find associations between mean tract-FA of the ventral pathways, including the left and right IFOF, ILF, and UF, and later reading outcome in either birth group. The distinct pattern of associations suggests that the neural basis of learning to read may be different in children born PT and FT. Variation in the neural substrates of reading may reflect the ability of the PT brain to recover from or compensate for neural consequences of PT birth, allowing these children to achieve reading skills within the normal range. The findings, thus, suggest that plasticity after white matter changes related to PT birth may be associated with changes in brain wiring supporting reading.

### White Matter Plasticity in Children Born PT

We know that children born PT are at risk for white matter injury (Back and Rosenberg, 2014; Back, 2017). Before significant advances in neonatal care, cystic lesions within the periventricular zone were the most common form of white matter injury during PT birth (Gano et al., 2015). Today, non-cystic lesions within similar white matter regions predominate (Back et al., 2007; Volpe, 2009). Imaging studies have established that children born PT show differences in microstructural properties of major white matter pathways compared to their FT peers in the neonatal period (Anjari et al., 2007; Giménez et al., 2008; Rose et al., 2008), and also persisting into childhood (Nagy et al., 2003; Andrews et al., 2010; Dodson et al., 2017), adolescence (Nagy et al., 2003; Vangberg et al., 2006; Groeschel et al., 2014; Travis et al., 2015a), and adulthood (Allin et al., 2011; Eikenes et al., 2011). Studies vary in terms of the direction of group differences, which may be the result of differences in study samples, imaging methods, or analytic strategies. In any case, at the level of neurobiology, we have yet to learn whether white matter differences between children born PT and FT represent downstream effects of injury, long-term consequences of white matter dysmaturity, or reorganization of the PT brain. Such insights cannot be determined directly from diffusion scans taken at a single point in time and will require longitudinal imaging studies.

At the behavioral level, children born PT as a group experience long-term decrements in reading, but generally score within the normal range on standardized tests of reading (Aarnoudse-Moens et al., 2009; Lee et al., 2011; Kovachy et al., 2015). These results are behavioral indications of neural plasticity. The magnitude of group differences has been found to decrease for simple language functions (van Noort-van der Spek et al., 2012), though not for complex language functions (van Noort-van der Spek et al., 2012) or reading (Kovachy et al., 2015).

Structural and functional imaging studies have implicated impaired connectivity as relevant to reading and language difficulties after PT birth (Gozzo et al., 2009; Northam et al., 2012). An alternative frame is to consider white matter differences as an index of neuroplasticity after early injury and/or dysmaturity (Sampaio-Baptista and Johansen-Berg, 2017). Mullen et al. (2011) found that receptive vocabulary and rapid naming skills were correlated with diffusion metrics obtained from bilateral dorsal pathways in their PT group, suggesting that the PT participants relied heavily on the right hemisphere pathways. The FT comparison group in that study showed no significant associations of white matter metrics and reading (Mullen et al., 2011). Constable et al. (2013) used functional connectivity to interrogate cerebellar-cerebral connections and found increased connectivity was associated with receptive vocabulary and verbal comprehension in the PT sample. Findings from both of these studies, as the authors stated, may represent either a delay in maturation of white matter microstructure in the PT sample or the engagement of alternative neural pathways for language in PT adolescents. Yeatman and Feldman (2013) were able to document the use of alternate pathways for language and reading in a child born PT. In this case, a 12-year old girl who had been born PT and diagnosed with periventricular leukomalacia, a condition characterized by severe damage to the white matter primarily surrounding the ventricles, was lacking the Arc and SLF bilaterally. Despite early language delays, the child achieved average scores on expressive language, sentence repetition, and reading (Yeatman and Feldman, 2013). Analyses suggested that she relied on intact ventral connections between the temporal and frontal lobes through the extreme capsule and UF rather than major dorsal pathways. All of these reports emphasize that white matter integrity is likely an important component of impaired performance and also a factor promoting plasticity in children born PT.

### White Matter Pathways Were Associated With Later Reading in Children Born FT

The association of mean tract-FA to later reading outcome in FT children is consistent with many other studies. In typically developing children, diffusion metrics of the left AF and left SLF have been associated with various reading skills across different ages (Yeatman et al., 2011; Vandermosten et al., 2012b; Myers et al., 2014; Gullick and Booth, 2015; Travis et al., 2016a) Both pathways are thought to represent the dorsal stream in cognitive models of language and reading, linking inferior frontal with superior temporal cortices (Hickok and Poeppel, 2004; Scott and Wise, 2004) which have been implicated in phonological awareness and other pre-literacy skills (Houdé et al., 2010).

The association of the right SLF with later reading is in line with studies of developmental reading disorders. Among children with a familial risk for dyslexia, those children who subsequently developed into good readers showed faster white matter development in the right SLF compared to those who developed into poor readers (Wang et al., 2017). In addition, Hoeft et al. (2011) demonstrated that diffusion metrics of the right SLF predicted future reading gains in children with dyslexia but not in typical readers. Both studies suggest that the development of the right SLF may be a potential compensatory mechanism for white matter alterations within the left hemisphere, allowing children who are at risk for dyslexia to achieve fluent reading (Hoeft et al., 2011; Wang et al., 2017).

We have demonstrated that the right SLF is also associated with later reading outcomes in FT children (Borchers et al., 2019a). Because the right SLF was not associated with language or phonological awareness skills in that study, we proposed that the associations may reflect other skills that are involved in learning to read, such as executive function skills (Blair and Razza, 2007; Frye et al., 2009).

Cerebellar pathways have been recently implicated in reading. Travis et al. (2015b) reported associations between diffusion metrics of the cerebellar peduncles and reading in older children and adolescents. We have also shown that FA of the left ICP at age 6 years makes important contributions to reading outcome at age 8 years, even after consideration of preliteracy skills (Borchers et al., 2019a). Together, these studies suggest that cerebellar pathways contribute to cognitive processes that are integral to reading development. Because the ICP mostly contains afferent fibers from the spine and the olivary nucleus to the cerebellum, and efferent fibers from the cerebellum to the vestibular nuclei (Naidich and Duvernoy, 2009), our results may seem somewhat surprising. However, proficient reading is likely to also depend on implicit learning and feedback processes which support the execution of new perceptual and motor skills, such as oculomotor control and articulation (Stein et al., 2000; Velay et al., 2002). The ICP could mediate these processes and thus facilitate the fine-tuning and automatization of core functions of reading.

### White Matter Pathways Were Not Associated With Later Reading in Children Born PT

In contrast to children born FT, we did not observe any significant associations between mean tract-FA of the dorsal or ventral pathways and later reading outcome in children born PT. While null findings are exceedingly common and represent potentially important discoveries, they are difficult to interpret. One possible explanation is that the size of our PT group may have been adequate only to detect very strong correlations at 5% alpha (two-tailed). Given that we had sufficient power to find significant associations in our FT group, however, we expected to also have sufficient power to find associations in the PT group, which was of similar size.

Another explanation for the lack of findings may be that children born PT rely on a different set of white matter pathways to achieve proficiency in reading accuracy, fluency, and comprehension. However, we did not find any associations with mean tract-FA of the left and right IFOF, ILF, and UF. Since we examined a comprehensive set of white matter pathways implicated in reading including dorsal, ventral, and cerebellar pathways, we think that this explanation is unlikely. Alternatively, children born PT may involve a larger network in which no specific pathway is predominant for reading. In a recent study, we assessed how different domains of cognitive function, often impacted by PT birth, were associated with reading outcome in children born PT relative to children born FT (Borchers et al., 2019b). While verbal skills (phonological awareness, language) at age 6 years were associated with reading outcome in both children born PT and FT, non-verbal cognitive skills (executive function, non-verbal IQ) were only associated with reading outcome in children born PT. These findings suggest that children born PT rely on a broader set of cognitive skills to achieve reading proficiency than children born FT (Borchers et al., 2019b). Whether this broader set of cognitive skills may in turn be mediated by a larger network of white matter pathways is an important topic for future research.

It is also possible that children born PT represent a heterogeneous group, both in terms of behavior and white matter injury. In this study, the sample of PT children did not differ from the sample of FT children either in reading outcome or mean tract-FA. In addition, the PT group was generally of high SES and had relatively strong IQ scores compared to other samples of PT children (Bhutta et al., 2002). Therefore, these findings may not be representative of PT children of low SES and/or with lower IQ scores. We recognize that associations of reading outcome and mean tract-FA of white matter pathways may be present in a subset of PT children. However, because our sample was moderate in size and relatively homogeneous, we were not able to consider subgroup analyses, such as comparing relations in extremely low gestational age (gestational age at birth less than 28 weeks) versus very low gestational age (gestational age at birth over 28 weeks).

Lastly, children born PT may rely on the same pathways as children born FT, but FA may be insensitive to their individual variation in reading outcome. FA is an indirect measure of white matter microstructure and influenced by a number of different tissue factors such as myelin thickness, axonal diameter, axon density, and crossing fibers (Beaulieu, 2002; Wheeler-Kingshott and Cercignani, 2009). Because hypoxia, ischemia, and inflammation associated with PT birth can affect white matter maturation (Back et al., 2007; Khwaja and Volpe, 2007; Volpe, 2009), FA may index a different or more diverse set of tissue factors in children born PT compared to children born FT. Ongoing studies are exploring these possibilities.

## Conclusion

Microstructural properties of cerebral and cerebellar white matter pathways at age 6 years were associated with reading outcome 2 years later in children born FT, but not in children born PT. Our findings suggest that the two groups may have important differences in the neural basis of reading development. These differences may point to white matter plasticity after injury in children born PT, given that they learn to read, often within the normal range. Overall, this study highlights that there may be multiple routes to learning to read. Design of behavioral assessment and intervention should consider birth group status. The metrics we have used here, namely FA, may not be specific enough to detect recovery of function and differential development in children born PT. Future studies should integrate diffusion MRI with imaging methods that can provide direct measures of myelin content and axonal diameter (e.g., Assaf et al., 2008; Mezer et al., 2013) to assess and triangulate the associations between white matter pathways and reading abilities in children born PT.

## Ethics Statement

This study was carried out in accordance with the recommendations of the Stanford University Institutional Review Board. In all cases, a parent or guardian provided written informed consent in accordance with the Declaration of Helsinki. The protocol was approved by the Stanford University Institutional Review Board (#IRB-22233).

## Author Contributions

LB analyzed the data and drafted the initial manuscript. LRB and CD collected and analyzed the data, reviewed, and revised the manuscript. VM, KT, and MB-S contributed to conceptualization of data analysis, coordinated and supervised data analysis, critically reviewed the manuscript for intellectual content, and revised the manuscript. HF conceptualized and designed the study, selected the data collection instruments, coordinated and supervised data collection, assisted in the creating of the initial draft of the manuscript, critically reviewed the manuscript for intellectual content, and revised the manuscript. All authors approved the final manuscript as submitted and agreed to be accountable for all aspects of the work.

## Conflict of Interest Statement

The authors declare that the research was conducted in the absence of any commercial or financial relationships that could be construed as a potential conflict of interest.

## Footnotes

1 https://github.com/vistalab/vistasoft/tree/master/mrDiffusion
