# Supplementary material for: White Matter Plasticity in Reading-Related Pathways Differs in Children Born Preterm and at Term: A Longitudinal Analysis
Source: Front Hum Neurosci. 2019 May 8;13:139. doi: 10.3389/fnhum.2019.00139 (PMC6519445; doi:10.3389/fnhum.2019.00139)
Supplement: Supplementary file 1 [file Table_1.DOCX]

Supplementary Material

White matter plasticity in reading-related pathways differs in children born preterm and at term: a longitudinal analysis

Lisa Bruckert, Lauren R. Borchers, Cory K. Dodson, Virginia A. Marchman, Katherine E. Travis, Michal Ben-Shachar, Heidi M. Feldman*

*** Correspondence:** Heidi M. Feldman, MD, PhD: [hfeldman@stanford.edu](mailto:hfeldman@stanford.edu)

**Table 1S.** Subgroup differences on reading outcome assessed using the Oral Reading Index (GORT-5) at age 8y (significant p-values are printed in bold).

| **Variable** | **Status** | ***n*** | ***M*** | ***SD*** | ***T-Test / Mann-Whitney U Test*** | | | |
| --- | --- | --- | --- | --- | --- | --- | --- | --- |
|  |  |  |  |  | *t* or *U* | *df* | *p* | *d* |
| **Sex** | Male | 37 | 96.7 | 9.11 | -2.43 | 69 | **.018** | 0.57 |
|  | Female | 34 | 103.6 | 14.2 |  |  |  |  |
| **Language status** | Monolingual | 41 | 98.7 | 11.0 | -1.08 | 69 | .284 | 0.25 |
|  | Bilingual | 30 | 101.8 | 13.8 |  |  |  |  |
| **Grade age 6y** | Kindergarten | 51 | 99.7 | 11.0 | 506.0 | 69 | .959 | 0.09 |
|  | 1^st^ Grade | 20 | 100.9 | 15.2 |  |  |  |  |
| **FH of reading delay** | Negative | 62 | 100.6 | 11.8 | 212.0 | 69 | .245 | 0.36 |
|  | Positive | 9 | 95.7 | 15.3 |  |  |  |  |

**Table 2S.** Pearson correlations between the reading outcome at age 8y and covariates, pre-literacy skills, and mean tract-FA of white matter pathways at age 6y. Data are Pearson correlation coefficients (df).

|  | **Reading**^1^  **outcome** | **SES** | **IQ** | **Language** | **Phono**  **Awareness** | **Arc-L** | **SLF-L** | **SLF-R** |
| --- | --- | --- | --- | --- | --- | --- | --- | --- |
| **SES**^2^ | 0.35^b^  (70) | - | - | - | - | - | - | - |
| **IQ**^3^ | 0.42^c^ (70) | 0.48^c^ (70) | - | - | - | - | - | - |
| **Language**^4^ | 0.60^c^ (70) | 0.55^c^ (70) | 0.61^c^ (70) | - | - | - | - | - |
| **Phono Awareness**^5^ | 0.54^c^ (70) | 0.23 (70) | 0.42^c^ (70) | 0.57^c^ (70) | - | - | - | - |
| **Arc-L**^6^ | 0.24^a^ (69) | 0.15 (69) | 0.00 (69) | 0.19 (69) | 0.34^b^ (69) | - | - | - |
| **SLF-L**^7^ | 0.35^b^ (70) | 0.07 (70) | 0.10 (70) | 0.16 (70) | 0.23^a^ (70) | 0.34^b^ (69) | - | - |
| **SLF-R**^7^ | 0.29^a^ (70) | 0.22 (70) | 0.02 (70) | 0.06 (70) | 0.14 (70) | 0.39^c^ (69) | 0.26^a^ (70) | - |
| **ICP-L**^8^ | 0.24 (66) | 0.13 (66) | -0.12 (66) | 0.11 (66) | -0.03 (66) | 0.19 (65) | 0.20 (66) | 0.27^b^ (66) |

^1^Oral Reading Index, measured with the GORT-5 measured at age 8y

^2^Socioeconomic status, measured with the Hollingshead Index measured at age 6y

^3^Non-verbal Intelligence Quotient, measured with the WASI-II measured at age 6y

^4^Core Language Index, measured with the CELF-4 measured at age 6y

^5^Phonological Awareness Composite, measured with the CTOPP measured at age 6y

^6^Arcuate Fasciculus

^7^Superior Longitudinal Fasciculus

^8^Inferior Cerebellar Peduncle

^a^*p* < .05, ^b^*p* < .01, ^c^*p* < .001

**Table 3S.** Pearson correlations between the reading outcome at age 8y and covariates, pre-literacy skills, and mean tract-FA of white matter pathways at age 6y. Data are Pearson correlation coefficients (df).

|  | **Reading**^1^  **outcome** | **SES** | **IQ** | **Language** | **Phono**  **Awareness** | **ILF-L** | **ILF-R** | **UF-L** |
| --- | --- | --- | --- | --- | --- | --- | --- | --- |
| **SES**^2^ | 0.35^b^  (70) | - | - | - | - | - | - | - |
| **IQ**^3^ | 0.42^c^ (70) | 0.48^c^ (70) | - | - | - | - | - | - |
| **Language**^4^ | 0.60^c^ (70) | 0.55^c^ (70) | 0.61^c^ (70) | - | - | - | - | - |
| **Phono Awareness**^5^ | 0.54^c^ (70) | 0.23 (70) | 0.42^c^ (70) | 0.57^c^ (70) | - | - | - | - |
| **ILF-L**^6^ | 0.03 (70) | 0.21 (70) | -0.08 (70) | 0.04 (70) | -0.03 (70) | - | - | - |
| **ILF-R**^6^ | 0.13 (70) | 0.10 (70) | -0.05 (70) | 0.08 (70) | 0.02 (70) | 0.61^c^ (70) | - | - |
| **UF-L**^7^ | -0.02 (70) | -0.04 (70) | 0.04 (70) | -0.01 (70) | -0.05 (70) | 0.03 (70) | -0.17 (70) | - |
| **UF-R**^7^ | 0.12 (70) | 0.01 (70) | -0.01 (70) | 0.17 (70) | 0.05 (70) | 0.17 (70) | 0.06 (70) | 0.72^c^ (70) |

^1^Oral Reading Index, measured with the GORT-5 measured at age 8y

^2^Socioeconomic status, measured with the Hollingshead Index measured at age 6y

^3^Non-verbal Intelligence Quotient, measured with the WASI-II measured at age 6y

^4^Core Language Index, measured with the CELF-4 measured at age 6y

^5^Phonological Awareness Composite, measured with the CTOPP measured at age 6y

^6^Inferior Longitudinal Fasciculus

^7^Uncinate Fasciculus

^a^*p* < .05, ^b^*p* < .01, ^c^*p* < .001

**Table 4S.** Prediction of reading outcome at age 8y by mean tract-FA of the left Arcuate (Arc-L), left and right superior longitudinal fasciculus (SLF-L, SLF-R) and left inferior cerebellar peduncle (ICP-L) at age 6y, controlling for sex, socio-economic status (SES) and non-verbal intelligence (IQ) in children born preterm and full term -- excluding children with a family history of reading delays.

|  | **Model 3A** | **Model 3B** | **Model 3C** | **Model 3D** | **Model 3E** |
| --- | --- | --- | --- | --- | --- |
| **Sex** | 1.0 (2.9) | 0.7 (2.8) | 0.1 (2.8) | 1.8 (2.6) | 0.3 (2.6) |
| **SES** | 0.2 (0.1) | 0.2 (0.1) | 0.2 (0.1) | 0.2 (0.1) | 0. (0.1) |
| **IQ** | 0.2 (0.1)^a^ | 0.3 (0.1)^b^ | 0.2 (0.1)^a^ | 0.3 (0.1)^b^ | 0.3 (0.1)^c^ |
| **Group** | -4.1 (2.9) | 98.9 (40.5)^a^ | 38.9 (27.0) | 75.0 (25.0)^b^ | 71.6 (26.5)^b^ |
| **Arc-L** | ­­- | 193.7 (65.7)^b^ | - | - | - |
| **Arc-L x birth group** | - | -210.8 (83.5)^a^ | - | - | - |
| **SLF-L** | - | - | 122.9 (47.8)^a^ | - | - |
| **SLF-L x birth group** | - | - | -102.2 (63.0) | - | - |
| **SLF-R** | - | - | - | 150.9 (36.3)^c^ | - |
| **SLF-R x birth group** | - | - | - | -166.4 (51.8)^b^ | - |
| **ICP-L** | - | - | - | - | 177.4 (43.6)^c^ |
| **ICP-L x birth group** | - | - | - | - | -173.8 (60.1)^b^ |
| **∆ R^2^** | - | 7.0%^a^ | 2.9% | 9.7%^b^ | 7.9%^b^ |
| **Total R^2^** | 31.5%^c^ | 40.6%^c^ | 39.1%^c^ | 48.0%^c^ | 51.8%^c^ |

Data are unstandardized coefficients (SE). ^a^*p* < .05, ^b^*p* < .01, ^c^*p* < .001

^*^R^2^ change (**∆** R^2^) values in models 3B-3E reflect the increase in variance accounted for by the interaction term in relation to a model with the main effect for that tract.

**Table 5S.** Prediction of reading outcome at age 8y by mean tract-FA of the left Arcuate (Arc-L), left and right superior longitudinal fasciculus (SLF-L, SLF-R) and left inferior cerebellar peduncle (ICP-L) at age 6y, controlling for sex, socio-economic status (SES), non-verbal intelligence (IQ), language, and phonological awareness (Phono awareness) in children born preterm and full term -- excluding children with a family history of reading delays.

|  | **Model 4A** | **Model 4B** | **Model 4C** | **Model 4D** | **Model 4E** |
| --- | --- | --- | --- | --- | --- |
| **Sex** | -1.8 (2.7) | -2.0 (2.6) | -2.3 (2.7) | -1.2 (2.3) | -1.5 (2.6) |
| **SES** | 0.1 (0.1) | 0.1 (0.1) | 0.1 (0.1) | 0.0 (0.1) | 0.1 (0.1) |
| **IQ** | 0.1 (0.1) | 0.1 (0.1) | 0.1 (0.1) | 0.1 (0.1) | 0.2 (0.1)^a^ |
| **Language** | 0.3 (0.2)^a^ | 0.4 (0.2)^a^ | 0.3 (0.2)^a^ | 0.4 (0.1)^c^ | 0.2 (0.2) |
| **Phono awareness** | 0.2 (0.1) | 0.2 (0.1) | 0.2 (0.1) | 0.2 (0.1) | 0.1 (0.1) |
| **Group** | -1.9 (2.8) | 96.6 (37.0)^a^ | 30.2 (25.0) | 91.1 (21.5)^c^ | 64.0 (26.5)^a^ |
| **Arc-L** | - | 164.1 (61.9)^a^ | - | - | - |
| **Arc-L x birth group** | - | -202.1 (76.3)^a^ | - | - | - |
| **SLF-L** | - | - | 97.5 (44.6)^a^ | - | - |
| **SLF-L x birth group** | - | - | -77.1 (58.5) | - | - |
| **SLF-R** | - | - | - | 158.5 (31.1)^c^ | - |
| **SLF-R x birth group** | - | - | - | -194.3 (44.5)^c^ | - |
| **ICP-L** | - | - | - | - | 158.8 (42.7)^c^ |
| **ICP-L x birth group** | - | - | - | - | -153.5 (59.9)^a^ |
| **∆ R^2^** | - | 6.4%^a^ | 1.6% | 13.0%^c^ | 5.7%^a^ |
| **Total R^2^** | 45.5%^c^ | 52.3%^c^ | 50.2%^c^ | 64.0%^c^ | 57.2%^c^ |

Data are unstandardized coefficients (SE). ^a^*p* < .05, ^b^*p* < .01, ^c^*p* < .001

^*^R^2^ change (**∆** R^2^) values in models 4B-4E reflect the increase in variance accounted for by the interaction term in relation to a model with the main effect for that tract.
